# Chimeric Peptide Tat-HA-NR2B9c Improves Regenerative Repair after Transient Global Ischemia

**DOI:** 10.3389/fneur.2017.00509

**Published:** 2017-09-26

**Authors:** Hai-Hui Zhou, Li Zhang, Hai-Xia Zhang, Jin-Ping Zhang, Wei-Hong Ge

**Affiliations:** ^1^Division of Clinical Pharmacy, Department of Pharmacy, Drum Tower Hospital Affiliated to Medical School of Nanjing University, Nanjing, China; ^2^Pharmacy Department, The First Affiliated Hospital, Nanjing Medical University, Nanjing, China

**Keywords:** transient global ischemia, hippocampus, PSD95, regenerative repair, spatial learning and memory

## Abstract

Transient global ischemia (TGI) is a major public health problem, and it heightens the need of effective treatments. The present study was undertaken to investigate whether recombinant polypeptide Tat-HA-NR2B9c improves spatial learning and memory deficits in rats after TGI. Rats were subjected to 20-min ischemia induced by four-vessel occlusion (4-VO) method and daily injected with Tat-HA-NR2B9c (1.12 mg/kg) for 1 week. Tat-HA-NR2B9c increased CREB activity, upregulated B-cell lymphoma-2 (Bcl-2) expression after treated for 24 h. There was a significant increase in dendrite spine density in hippocampal CA1 region and BrdU-positive cells and BrdU/NeuN-positive cells in the dentate gyrus after Tat-HA-NR2B9c treatment, compared with ischemia group at postischemic day 28. Inhibition of the CREB activation by recombinant lentivirus, LV-CREB133-GFP, abolished the upregulation effects of Tat-HA-NR2B9c on Bcl-2 expression. Moreover, Tat-HA-NR2B9c improved the impaired spatial learning and memory ability in Morris water maze. These results suggest that Tat-HA-NR2B9c substantially ameliorated the TGI-induced loss of dendrite spine in hippocampal CA1, increased neurogenesis in dentate gyrus, and significantly improved cognitive abilities by the CREB pathway in rats after transient global cerebral ischemia. It may be served as a treatment for TGI.

## Introduction

Transient global ischemia (TGI) occurs when blood flow stops or reduced after cardiac and respiratory arrest, and leads to neural dysfunction and neuron necrosis in hippocampus, especially the CA1 pyramidal neurons (1). Delayed neuronal death often happens on 3–4 days after the onset of ischemic injury (2, 3), and many of the newborn hippocampal neurons die within a few weeks (4). Excessive release of glutamate causes rise to ion channel receptor activation and in turn recruits intracellular multiprotein signaling complexes by the postsynaptic density (PSD) (5, 6). A prominent organizing protein is PSD-95, which binds both *N*-Methyl-d-aspartate receptors (NMDARs) and neuronal nitric oxide synthase (nNOS) at excitatory synapses (7, 8). NMDARs play an important role in synaptic plasticity, thus blocking them is deleterious (9, 10). Targeting PSD-95 protein, therefore, represents an alternative therapeutic strategy for TGI. Recently, we prepared a recombinant chimeric peptide Tat-HA-NR2B9c, which contains last nine amino acids of the carboxyl tail of GluN2B, an influenza virus hemagglutinin epitope-tag (HA) and the 11-mer Tat protein transduction domain of the human immunodeficiency virus-type 1. We found that Tat-HA-NR2B9c reduced infarct volume and improved nerve defect symptoms in rats subjected to middle cerebral artery occlusion (MCAO) by treatment within 1 h after reperfusion (11). Moreover, in 2015, delayed treatment with Tat-HA-NR2B9c promotes recovery after MCAO (12). However, it remains unknown whether Tat-HA-NR2B9c improves TGI outcome and its mechanism. Uncoupling GluN2B-PSD95 could decrease NO production (6), and nNOS-derived NO exerts a negatively control on the adult neurogenesis in normal and ischemic brains *in vivo* (13, 14). What is more, nNOS-derived NO negative control CREB activity (15). In brain, CREB-mediated gene transcription is involved in memory, learning, and synaptic transmission (16). Activation of CREB leads to upregulation of survival gene such as B-cell lymphoma-2 (Bcl-2) (17). Thus, Tat-HA-NR2B9c may increase gene transcription of upregulating Bcl-2 through phosphorylation of CREB. Here, we investigated the regenerative repair effects of Tat-HA-NR2B9c on TGI and reported that Tat-HA-NR2B9c improves TGI outcome through CREB-mediated upregulation of Bcl-2.

## Materials and Methods

### Drugs

Tat-HA-NR2B9c was designed and synthesized in our laboratory (11). It was dissolved in normal saline and intravenous (1.12 mg/kg/day, i.v.) injected. The injection volume was 3 ml/kg. Sham and vehicle groups were treated with corresponding volume of solvent.

### Animals

The Institutional Animal Ethical Committee of Nanjing University (Approval No., GY20160108) authorized the experimental protocol, and all procedures involving animals were performed in accordance with National Institute of Health guidelines for the care and use of laboratory animals. In this study, adult male Sprague-Dawley rats (250–300 g; B&K Universal Group Limited, Shanghai) were used. An experimenter labeled all animals before allocation. Experiments were performed by investigators who were blinded to group allocation.

### Surgical Preparation

Transient global ischemia was carried out using 4-VO model (1). On the first day, rats were anesthetized by 10% chloral hydrate (300 mg/kg, i.p.) and then fixed by stereotaxic ear bars. The head was placed at about 30° to the horizontal. After disinfection using 75% alcoholic, both the vertebral arteries were occluded permanently by electrocauterization with a 0.5-mm diameter needle through the foramen of the first cervical vertebra. The rats were kept recovering for 24 h. The next day, animals were reanesthetized and both common carotid arteries (CCAs) were occluded for 20 min using microvascular clips to induce cerebral ischemia. Then, both clips were softly removed in order to reperfusion. During the whole operation and in the following 6 h, body temperature was maintained at 37 ± 0.5°C with a thermostatically controlled infrared lamp. Any rat returned to righting reflexes during the 20 min occlusion or epileptic seizure after ischemia was excluded. Animals in sham group were treated with anesthetics two times and for the same period of time as the surgical animals without occluding CCAs.

### Morris Water Maze Task

The protocol of Morris water maze task for rats has been detailedly described in our previous study (18). During the visible platform trials, rats were placed in the opaque water of the circular swimming pool (Jiliang Neuroscience Inc., Shanghai) measuring 180 cm in diameter. Four starting points around the edge of the pool were designed as N, E, S, and W, which divided the pool into four quadrants. A 10 cm diameter was located in a contrast position in the middle of one quadrant. The location of the visible platform varied for each trial. Four trials were administered. The latency to reach the visible platform and swimming speed were measured. Then, rats were trained to locate the hidden platform 1.2 cm under the surface of the water. The task for the rats was to escape from the water by locating the hidden platform. In the training to find the hidden platform, rats were allowed to swim for maximum of 60 s in the pool for each trail. One block of four trials per day was given for five consecutive days. For each trail, the rats were placed in the water facing the wall of the pool. Each trial was videotaped *via* a ceiling-mounted video camera and the animal’s movement was tracked using Ethovision software (Noldus Information Technology), which allows the calculation a series of parameters such as latency. Next day, rats were given one 60-s retention probe test in which the platform was removed from the pool. During retention, the time spent in the target quadrant and the number of crossing of the platform location was measured.

### Golgi-Cox Staining

Golgi-Cox staining was performed with FD Rapid Golgi Stain TM Kit (FD NeuroTechnologies) as we previously reported (18). The rats were deep anesthetized by 10% chloral hydrate and killed on day 28 after TGI. The fresh brains were sliced into 10 mm thickness and placed in impregnation solution at room temperature. After 2 weeks, brains were transferred into Solution C and stored at 4°C in the dark for at least 48 h. Then, brains were cut into 100 µm coronal sections by vibratome (World Precision Instruments) and stained. For morphological analysis, 10 neurons randomly for each sample were measured and the average was regarded as the final value of one sample.

### Immunofluorescence

Immunofluorescence was performed as we previously reported (18). Animals were deep anesthetized by 10% chloral hydrate and perfused transcardially with 0.05 M sodium phosphate (pH 7.4) containing 0.8% NaCl, followed by 4% paraformaldehyde in 0.05 M sodium phosphate (pH 7.4, containing 0.8% NaCl). Brains were removed and postfixed overnight in the same solution. Serial hippocampal sections (40 µm) were made on an oscillating tissue slicer in a bath of physiological saline. The sections were heated (85°C for 5 min) in antigen-unmasking solution (Vector Laboratories); incubated in 2 M HCl (37°C for 30 min); rinsed in 0.1 M boric acid, pH 8.5, for 10 min; and blocked in PBS containing 3% normal goat serum, 0.3% (w/v) Triton X-100, and 0.1% bovine serum albumin at room temperature for 1 h, followed by incubation with primary antibodies at 4°C overnight. The primary antibodies used were as follows: rat anti-BrdU (1:200; Accurate Chemical & Scientific Corporation), mouse anti-NeuN (1:500; Millipore Bioscience Research Reagents). Subsequently, the sections were incubated with secondary antibodies goat anti-rat Cy3 (1:200; Millipore Bioscience Research Reagents), goat anti-mouse dylight 488 (1:400; Jackson ImmunoResearch). Appropriate horseradish peroxidase-linked secondary antibodies were used for detection by enhanced chemiluminescence (Pierce). An experimenter coded all slides from the experiments before quantitative analysis. To determine the total number of BrdU^+^/NeuN^+^ cells, the counts from sampled five sections were averaged, and the mean values were multiplied by the total number of sections. We used a confocal laser-scanning microscope (LSM700, Zeiss) to capture images and confirm colocalization of BrdU^+^/NeuN^+^ cells.

### Culture of Hippocampal Neurons

Culture of hippocampal neurons was performed as previously described (15). Primary neurons were isolated from E18 Sprague-Dawley rat hippocampus and cultured on dishes coated with polyornithine (10 µg/ml; Sigma-Aldrich) in Neurobasal medium (Invitrogen) containing 2% B27 supplement. The planting density 1 × 10^5^ cells/cm^2^ for biochemical detection. Cell cultures were kept in a humidified atmosphere of 95% air and 5% CO_2_ at 37°C. Half of the medium was replaced with fresh medium without glutamate every 2–3 days.

### Lentivirus Production and Infection of Neurons

A recombinant lentivirus, LV-CREB133-GFP, was generated with the plasmid pCMV-CREB133 (Clontech Laboratories, Mountain View, CA, USA) as we previous reported (14). LV-CREB133-GFP or LV-GFP (control LV) were added into cultured neurons at 5 μl/dish (diameter 3.5 cm). Twenty-four hours later, the medium was fully changed with fresh medium without lentivirus. After infected with the recombinant lentivirus for 5 days, neurons were passaged for experiments. The procedures concerning recombinant lentivirus were performed following National Institutes of Health guidelines.

### Oxygen Glucose Deprivation (OGD)

Oxygen glucose deprivation was performed as previously described with slight modification (19). Briefly, the primary hippocampal neurons were washed with glucose-free DMEM (Gibco, USA). Then, after the addition of glucose-free DMEM, they were placed in an anaerobic chamber containing 5% CO_2_ and 95% N_2_ at 37°C. We sealed cultures inside a modular chamber (Billups-Rothenberg) flushed for 10 min with the same premixed gas and placed inside an incubator for 3 h. OGD was terminated by replacing the exposure medium with neuronal culture medium added with Tat-HA-NR2B9c 250 nM, and returning the cells to a normoxic incubator to allow reperfusion for another 24 h. The cells cultured in the plain medium with ambient oxygen served as control (no exposure to OGD).

### Hoechst 33258 Staining

The cell viability was evaluated by using Hoechst 33258 (Sigma-Aldrich) nuclear staining as described previously (20), imaged with a fluorescence microscope (Axio Imager, Zeiss, Oberkochen, Germany), and analyzed with Image-Pro Plus software (Media Cybernetics, Silver Spring, MD, USA).

### Western Blot Analysis

Western bolt analysis was performed as we previously reported (18). Primary antibodies were rabbit antiphospho-CREB-ser133 (1:1,000; Cell signaling Technology); rabbit anti-CREB (1:1,000; Cell signaling Technology); rabbit anti-Bcl-2 (1:1,000; Cell Signaling Technology). Internal control was mouse anti-β-actin (1:1,000; Sigma-Aldrich). Appropriate horseradish peroxidase-linked secondary antibodies were used for detection by enhanced chemiluminescence (Pierce).

### Statistical Analyses

Statistical analysis for escape latency in the MWM test: repeated measured ANOVA followed by a *post hoc* Bonferroni multiple comparison test. Comparisons among multiple groups were made with one-way ANOVA (one factor) or two-way ANOVA (two factors) followed by Scheffe *post hoc* test. Data were presented as mean ± SEM and *P* < 0.05 was considered statistically significant. Investigators were blind to the group allocation when assessing the outcome.

## Results

### Tat-HA-NR2B9c Improves Spatial Memory in 4-VO Rats

**Figure 1:**
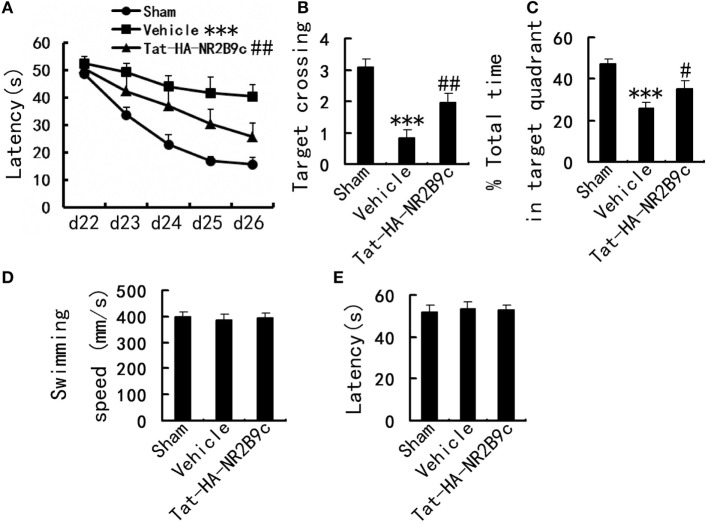
Effects of Tat-HA-NR2B9c on transient global ischemia (TGI)-induced spatial learning and memory deficits in rats as measured by Morris water maze. **(A)** Escape latency measured during days 22–26. **(B)** Target crossings; **(C)** time spent in target quadrant measured at day 27 after TGI in Morris water maze task. **(D)** Swimming speed; **(E)** the latency to reach platform in visible platform trials measured at day 21 after TGI (*n* = 10 for sham; *n* = 11 for vehicle, and Tat-HA-NR2B9c). Data are mean ± SEM (***P < 0.001 vs sham; ^#^*P* < 0.05, ^##^*P* < 0.01 vs vehicle).

To examine whether Tat-HA-NR2B9c benefits TGI outcome, we subjected rats to 4-VO and treated them with Tat-HA-NR2B9c (1.12 mg/kg i.v.) for 7 days starting at reperfusion. Spatial cognitive performance in Morris water maze was measured during days 21–27 after TGI. Ischemia significantly increased escaping latency, decreased time spent in target quadrant and target crossings in Morris water maze (Figures 1A–C), compared with sham, suggesting impaired spatial memory. Tat-HA-NR2B9c significantly ameliorated the ischemia-induced impairments of spatial memory in latency (Figure 1A); in target crossings (Figure 1B); and in total time in target quadrant (Figure 1C) compared with vehicle. The swimming speed (Figure 1D) and latency to reach platform (Figure 1E) were measured at day 21 in visible platform trials, Tat-HA-NR2B9c have no effect on cued behavior and gross motor skills of animals (Figures 1D,E).

### Tat-HA-NR2B9c Increases Neurogenesis in DG and Ameliorates Hippocampal CA1 Neuronal Damage after TGI

**Figure 2:**
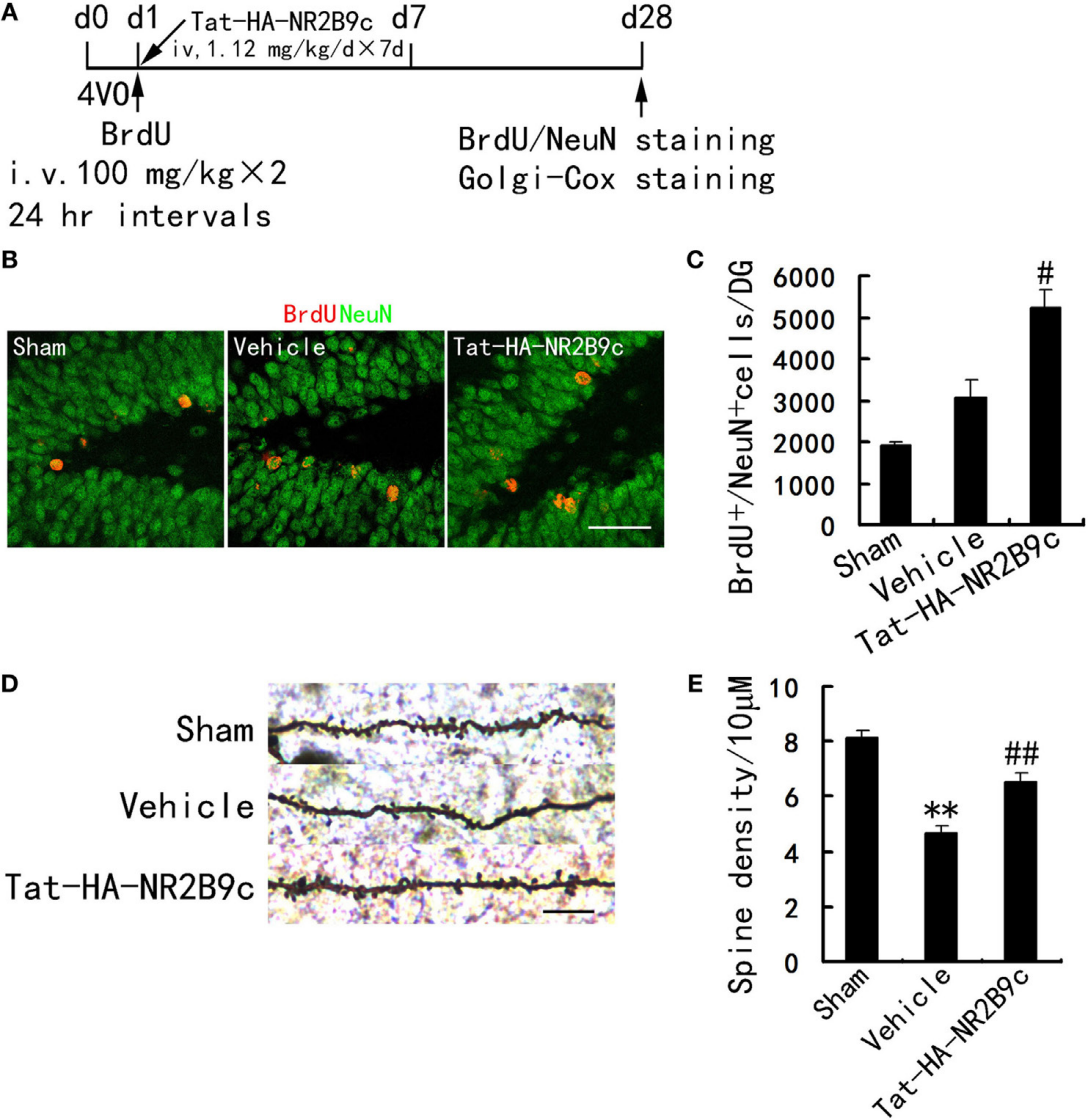
Tat-HA-NR2B9c increases neurogenesis in DG and ameliorates dendrite spine loss of pyramidal neurons in hippocampus CA1 region after transient global ischemia (TGI). **(A)** Schematic representation of experimental design for **(B–E)**. **(B,C)** Images and bar graph showing BrdU^+^/NeuN^+^ cells in dentate gyrus at day 28 after TGI (*n* = 7 for all groups). Scale bar, 50 µm. **(D,E)** Images and bar graph showing dendrite spine density in hippocampus CA1 region in rats subjected to TGI and treated with Tat-HA-NR2B9c (*n* = 5 for all groups; eight neurons were measured in each biological sample). Scale bar, 10 µm. Data are mean ± SEM (**P < 0.01 vs sham; ^#^*P* < 0.05, ^##^*P* < 0.01 vs vehicle).

Transient global ischemia leads to selective delayed neuronal death of pyramidal neurons in hippocampus CA1. We investigated whether Tat-HA-NR2B9c increased neurogenesis in dentate gyrus and reduced the apoptosis of pyramidal neurons in hippocampus CA1 region. Tat-HA-NR2B9c was administered for 7 days beginning immediately after reperfusion. Rats were treated with BrdU (100 mg/kg × 2 i.v.; 24-h intervals) to label proliferating cells during days 1–2 after TGI. These rats were killed at day 28 after TGI to estimate the number of BrdU^+^ and BrdU^+^/NeuN^+^ (a marker for mature neurons) cells (Figure 2A). Treatment with Tat-HA-NR2B9c significantly increased neurogenesis in hippocampus dentate gyrus (Figures 2B,C) and reversed the ischemia-induced dendrite spine loss of pyramidal neurons in hippocampus CA1 region compared with vehicle (Figures 2D,E).

### Tat-HA-NR2B9c Increases CREB Activity, Elevates Bcl-2 Expression in Hippocampal CA1 Region

**Figure 3:**
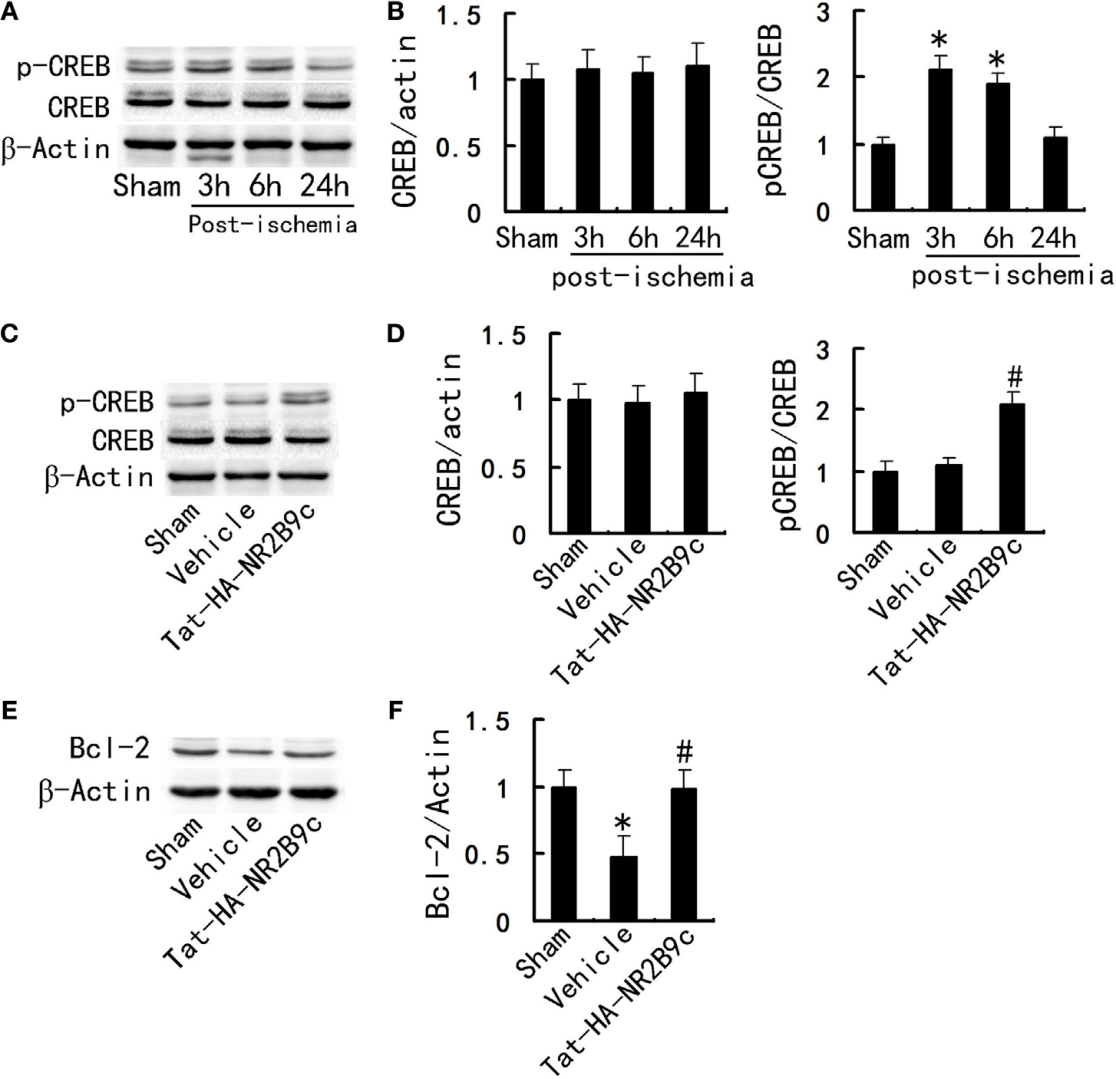
Tat-HA-NR2B9c increases the phosphorylation of CREB and elevates B-cell lymphoma-2 (Bcl-2) expression in the hippocampal CA1 region after transient global ischemia (TGI). **(A)** Immunoblots of phosphor-CREB (p-CREB) and CREB levels at serial time points following ischemia. **(B)** Statistical graph from data in **(A)**, *n* = 5. **(C)** Immunoblots of CREB, p-CREB in the CA1 region of rats treated with vehicle or Tat-HA-NR2B9c for 24 h following TGI. **(D)** Statistical graph from data in **(C)**, *n* = 5. **(E)** Immunoblots of Bcl-2 in the hippocampal CA1 region of rats treated with vehicle or Tat-HA-NR2B9c for 24 h following TGI. **(F)** Statistical graph from data in **(E)**, *n* = 5. Data are mean ± SEM (*P < 0.05 vs sham; ^#^*P* < 0.05 vs vehicle).

What is the underlying mechanism by which Tat-HA-NR2B9c improves the outcome of TGI? CREB is involved in the regulation of dendritic spine growth, neurogenesis, and synaptic plasticity. Activation of CREB leads to expression of genes such as Bcl-2 (17). It is well accepted that nNOS-derived NO exerts a negative control on the adult neurogenesis in normal and ischemic brains *in vivo* (14). NO negative control CREB activation (15), we thus investigated whether Tat-HA-NR2B9c affects CREB activation and expression of Bcl-2 after ischemia. To determine CREB activity, we examined phosphorylated CREB at Ser-133 (p-CREB-S133), an activated form of CREB. We subjected rats to TGI and measured levels of p-CREB, CREB, Bcl-2 at day 1 after TGI in hippocampus CA1 region. TGI caused a significant increase in CREB activity at 3 and 6 h postischemia and restore physiological levels at 24 h postischemia (Figures 3A,B). Tat-HA-NR2B9c increased the phosphorylation of CREB (Figures 3C,D) and elevated Bcl-2 (Figures 3E,F) expression in the hippocampal CA1 region at 24 h following global ischemia.

### Hippocampal CREB Phosphorylation Is Essential for the Regulation of Tat-HA-NR2B9c on Bcl-2

**Figure 4:**
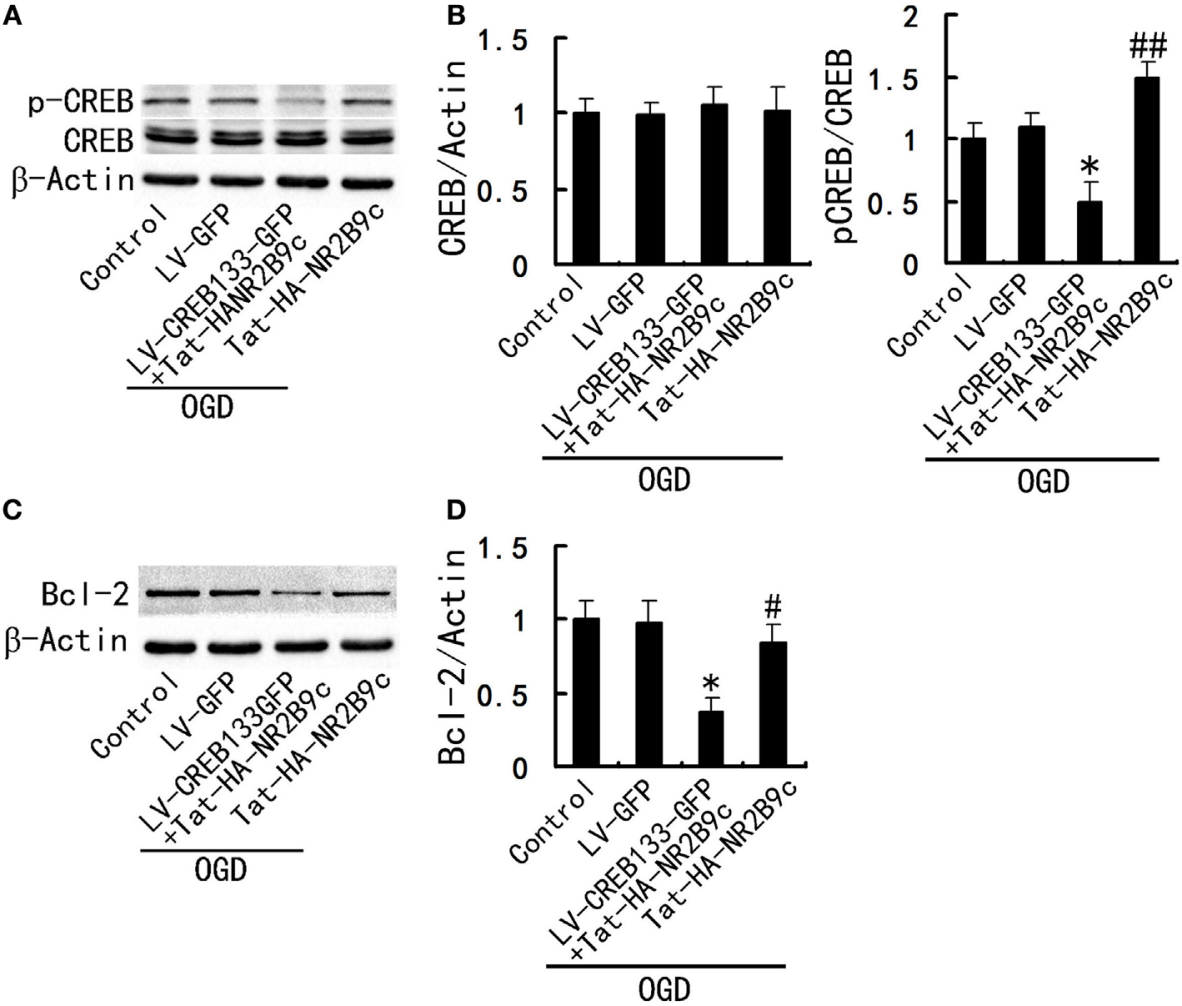
Inhibition of CREB activity abolish the regulation of Tat-HA-NR2B9c on B-cell lymphoma-2 (Bcl-2). **(A)** Immunoblots image showing phospho-CREB (p-CREB) and CREB in primary hippocampal neurons cultured in the plain medium (control); LV-GFP infected neurons; and neurons infected with LV-CREB133-GFP or not, exposed to oxygen glucose deprivation (OGD) and then treated with 250 nM of Tat-HA-NR2B9c, respectively. **(B)** Statistical graph from data in **(A)**, *n* = 5. **(C)** Immunoblots image showing Bcl-2 in primary hippocampal neurons cultured in the plain medium (control); LV-GPF infected neurons; and neurons infected with LV-CREB133-GFP or not, exposed to OGD and then treated with 250 nM of Tat-HA-NR2B9c, respectively. **(D)** Statistical graph from data in **(C)**, *n* = 5. Data are mean ± SEM (*P < 0.05 vs sham; ^#^*P* < 0.05 and ^##^*P* < 0.01 vs vehicle).

**Figure 5:**
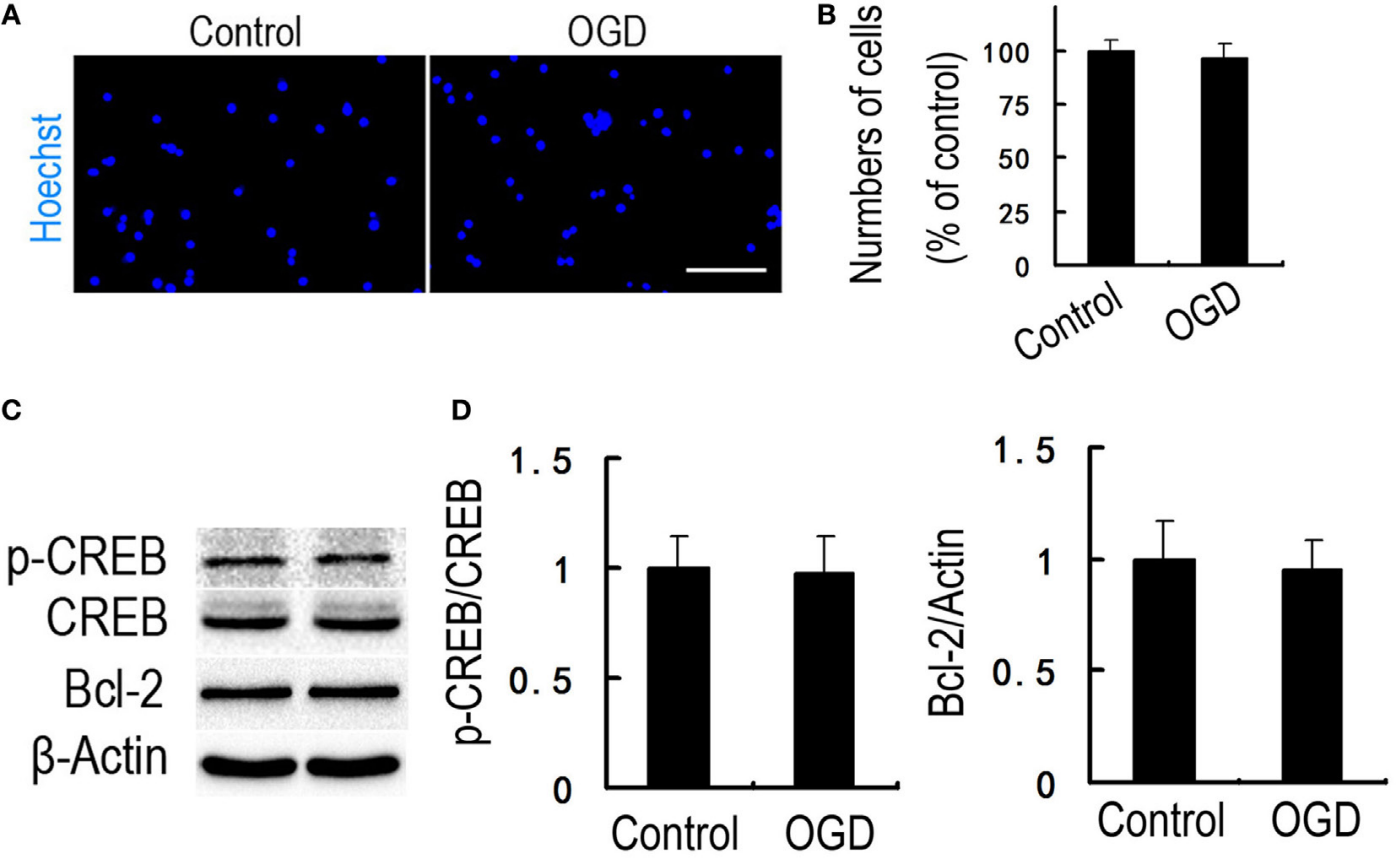
There are not differences in neuronal viability immediately after oxygen glucose deprivation (OGD). **(A)** Immunofluorescence of viable neurons. Nuclei were stained with Hoechst 33258 (blue). **(B)** Statistical graph from data in **(A)**, *n* = 5. **(C)** Immunoblots image showing phospho-CREB (p-CREB) and B-cell lymphoma-2 (Bcl-2) in primary hippocampal neurons cultured in the plain medium (control) or exposed to OGD. **(D)** Statistical graph from data in **(C)**, *n* = 5. Data are mean ± SEM. Scale bars = 50 µm **(A)**.

To determine whether hippocampal CREB activity is essential for the regulation of Tat-HA-NR2B9c to Bcl-2, we incubated the cultured hippocampal neurons with LV-CREB133-GFP, a recombinant lentivirus expressing a mutant variant of CREB protein, which could not be phosphorylated at Ser133. Tat-HA-NR2B9c activated phosphorylation of CREB (Figures 4A,B) and increased Bcl-2 expression (Figures 4C,D) in primary hippocampal neurons exposed to OGD 24 h later. LV-CREB133-GFP infection abolished Tat-HA-NR2B9c’s regulating on CREB activation (Figures 4A,B), and expression of Bcl-2 (Figures 4C,D). These findings indicate that hippocampus CREB mediated Tat-HA-NR2B9c regulation on Bcl-2. We also employed for the assessment of the numbers of viable cells, CREB phosphorilation, and Bcl-2 expression immediately after OGD. We found that there are not differences in neuronal viability immediately after OGD (Figure 5). This result suggested that the differences observed in these two proteins in Figure 4 are not due to different levels of neuronal death.

## Discussion

Disrupting NMDAR-PSD-95 interaction could protect neurons against NMDAR-mediated excitotoxicity without affecting calcium influx and NMDAR function (6, 7). In 2008, it was reported that intravenously using chemosynthetic peptide Tat-NR2B9c 1–3 h after stroke in rats reduced infarct volume and improved neurological outcome (21). Chemical synthesis of peptide, though very efficient, is a complex and costly process (22). So, it is not an ideal strategy for large-scale peptide production. We prepared a recombinant Tat-HA-NR2B9c peptide by genetic engineering techniques (11). Tat-HA-NR2B9c improve stroke outcome in the subacute phase (12). All the previous study employed focal cerebral ischemia models. Focal cerebral ischemia mainly damage cerebral cortex and striatum, the damage to motor function is the most obvious; whereas, TGI mainly damages the hippocampus, especially the CA1 region. The etiology and pathogenesis may make differences.

**Figure 6:**
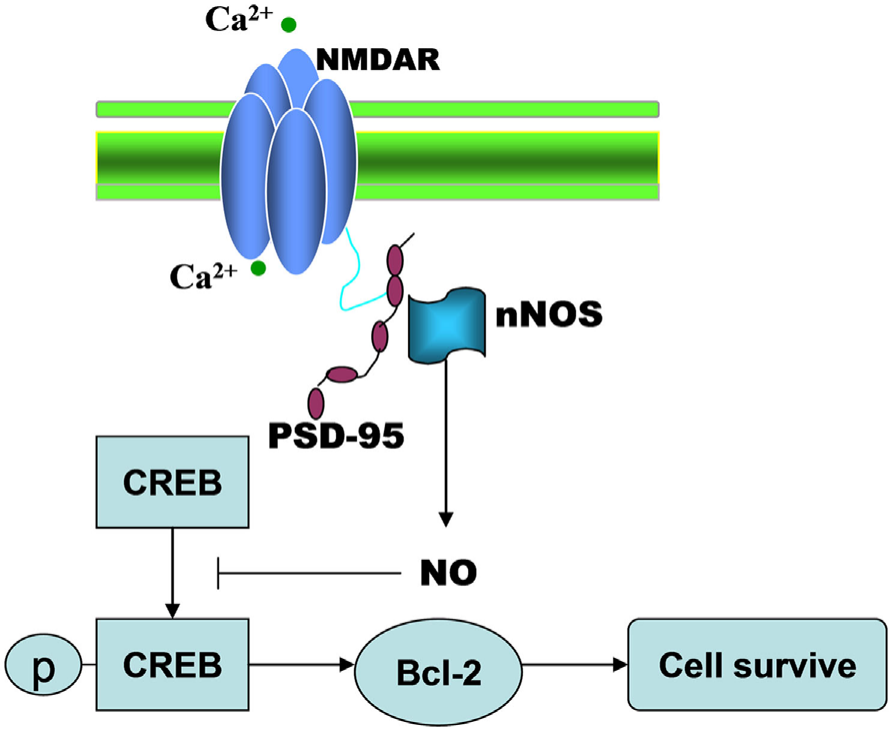
The illustration showing that TGI-induced NMDAR-PSD95 interaction negatively regulates regenerative repair in ischemic brain.

In the present study, we have identified a signaling cascade whereby NMDAR–PSD-95 interaction regulates TGI-induced impairments (Figure 6). Tat-HA-NR2B9c improved TGI-induced impairments of spatial memory by decreasing dendrite spine loss of hippocampal CA1 neurons and increasing neurogenesis in dentate gyrus induced by TGI in rats.

Neuroprotection and neurogenesis contribute to brain self-repair, thereby improving stroke outcome (23–25). Newborn CA1 granule cells are formed from precursor cells in the DG and SVZ areas that migrate and differentiate into neural cells of the CA1 (26). It has been established that nNOS-derived NO exerts a negative control on the adult neurogenesis in normal and ischemic brains *in vivo* (13). Indeed, the current study showed that dissociating NMDAR and PSD-95 enhances the survival and neuronal differentiation of NSCs, promotes dendritic spine formation in mature neurons in hippocampus CA1 region (Figure 2).

After infarction, neural dysfunctions such as hemiparesis and speech disturbance may improve during rehabilitation. CREB and CRE-mediated system are important for synaptic plasticity, neurogenesis and axon growth (27). CREB activation leads to expression of genes encoding neuroprotective molecules, such as the antiapoptotic protein Bcl-2, and contributes to survival of neurons (28). Tat-HA-NR2B9c increased the activity of CREB, expression of Bcl-2 in hippocampus CA1 region (Figure 3), thereby improves TGI-induced impairments of spatial memory (Figure 1). Hippocampal CREB phosphorylation is essential for the regulation of Tat-HA-NR2B9c on Bcl-2 (Figure 4).

In conclusion, the present study examined the effect of Tat-HA-NR2B9c in the rat model of TGI. Our results suggest that disrupting NMDAR–PSD-95 improves spatial memory after TGI. Tat-HA-NR2B9c increases neurogenesis in DG and ameliorates dendrite spine loss in hippocampal CA1 neuronal after TGI through increasing the phosphorylation of CREB and upregulating Bcl-2. This new drug may serve as a treatment for TGI.

## Ethics Statement

The Institutional Animal Ethical Committee of Nanjing University (Approval No., GY20160108) authorized the experimental protocol, and all procedures involving animals were performed in accordance with National Institute of Health guidelines for the care and use of laboratory animals.

## Author Contributions

H-HZ contributed to the design of the study, performed the surgical preparation and data analysis. LZ contributed to the western blotting, surgical preparation. H-XZ participated in behavioral analyses. J-PZ performed histochemical study. W-HG participated in the design of the studies.

## Conflict of Interest Statement

The authors declare that the research was conducted in the absence of any commercial or financial relationships that could be construed as a potential conflict of interest.
